# Prognostic Scoring System for Pulmonary Metastasectomy in Colorectal Cancer: External Validation and Clinical Implications for Adjuvant Chemotherapy

**DOI:** 10.3390/cancers18132072

**Published:** 2026-06-25

**Authors:** Hikaru Takahashi, Yoshikane Yamauchi, Tomoki Nishida, Masahiro Yanagiya, Hiroshi Hashimoto, Mingyon Mun, Yoko Azuma, Takekazu Iwata, Makoto Endo, Tomohiko Iida, Haruhisa Matsuguma, Takahiko Oyama, Takashi Ohtsuka, Yukinori Sakao

**Affiliations:** 1 Department of Surgery, Teikyo University School of Medicine, Tokyo 173-8605, Japan; htkhs009sur@gmail.com (H.T.); nishidatomoki.322@gmail.com (T.N.); ysakao070@gmail.com (Y.S.); 2 Department of Thoracic Surgery, The University of Tokyo Graduate School of Medicine, Tokyo 113-8655, Japan; yanagiyam-sur@h.u-tokyo.ac.jp; 3 Department of Thoracic Surgery, National Defense Medical College, Tokorozawa 359-8513, Japan; hashimoh@ndmc.ac.jp; 4 Department of Thoracic Surgical Oncology, The Cancer Institute Hospital, Tokyo 135-8550, Japan; mingyon.mun@jfcr.or.jp; 5 Division of Chest Surgery, Department of Surgery, Toho University School of Medicine, Tokyo 143-8541, Japan; yoko.azuma@med.toho-u.ac.jp; 6 Division of Thoracic Surgery, Chiba Cancer Center, Chiba 260-8717, Japan; tiwata@chiba-cc.jp; 7 Department of Thoracic Surgery, Yamagata Prefectural Central Hospital, Yamagata 990-2292, Japan; m-endoh@ypch.gr.jp; 8 Department of Thoracic Surgery, Kimitsu Central Hospital, Kimitsu 292-8535, Japan; hptiida@hotmail.co.jp; 9 Division of Thoracic Surgery, Tochigi Cancer Center, Utsunomiya 320-0834, Japan; hmatsugu@tochigi-cc.jp; 10 Department of General Thoracic Surgery, National Hospital Organization Tokyo Medical Center, Tokyo 152-8902, Japan; ot3465@gmail.com; 11 Division of Thoracic Surgery, Department of Surgery, Jikei University School of Medicine, Tokyo 105-8471, Japan; t-oh@remus.dti.ne.jp

**Keywords:** colorectal cancer, pulmonary metastasectomy, prognostic scoring, adjuvant chemotherapy, risk stratification

## Abstract

**Background**: Predicting long-term outcomes after pulmonary metastasectomy for colorectal cancer remains challenging because existing prognostic methods lack precision. We developed and validated a prognostic scoring system derived from a major international meta-analysis to improve risk stratification and to evaluate the benefit of adjuvant chemotherapy across risk groups. **Methods**: Using a Japanese registry of 819 patients who underwent lung resection between 2010 and 2019, we constructed a 0–13-point score based on eight variables including tumor size, number, biological markers, and intrathoracic lymph node status, which may require intraoperative or pathological confirmation. Granular data on chemotherapy regimens, timing, and duration were unavailable. Patients were classified as low, intermediate, or high risk. The primary analysis used inverse probability of treatment weighting to adjust for baseline imbalances; however, only 819 of 1657 patients (49.4%) had complete prognostic data, introducing potential selection bias. **Results**: The score separated patients into three groups with distinct five-year survival rates: 81.1% (low), 67.8% (intermediate), and 59.1% (high). In high-risk patients, chemotherapy was associated with improved overall survival but did not delay recurrence. In low-risk patients, chemotherapy correlated with reduced recurrence-free survival, a finding that persisted after adjustment. **Conclusions**: This validated scoring system aids individualized surgical decision making by identifying patients unlikely to benefit from routine postoperative chemotherapy. Observed survival advantages in high-risk patients may reflect selection of fitter individuals rather than direct treatment effects, underscoring the need to address selection bias in future trials.

## Simple Summary

Predicting outcomes after pulmonary metastasectomy for colorectal cancer remains challenging, as existing prognostic factors lack the precision required for optimal patient selection. This study developed and validated a novel prognostic scoring system based on a landmark meta-analysis to improve risk stratification and guide post-operative chemotherapy decisions. The resulting system effectively categorized patients into three distinct risk groups. Our retrospective analysis highlights that routine chemotherapy may not benefit low-risk patients and was associated with earlier recurrence. Additionally, survival benefits in high-risk patients were not accompanied by improved recurrence-free survival, suggesting they may be attributable to patient selection rather than a direct treatment effect. These findings provide a practical, evidence-based framework for individualized surgical decisions and may significantly influence the design of future clinical trials by addressing selection biases in treatment efficacy.

## 1. Introduction

Colorectal cancer (CRC) is the third most common malignancy and the second leading cause of cancer-related deaths worldwide, accounting for approximately 10% of all cancer diagnoses [1,2]. Metastatic disease develops in approximately 50% of CRC patients [3]. The lung is the second most frequent site of metastasis after the liver, accounting for 5% to 15% of all CRC cases [4,5]. Notably, lung metastases are more common in rectal cancer (approx. 5.6%) than in colon cancer (approx. 3.7%) [6,7]. This is attributed to the anatomical difference in venous drainage, where rectal cancer cells can enter the vena cava directly through the inferior and middle rectal veins, bypassing the portal system and the liver [8,9]. The presence of metastasis is the primary determinant of long-term survival and is responsible for 90% of all tumor-related deaths in CRC [10]. The 5-year survival rate, which is approximately 56% for non-metastatic cases, drops significantly once metastasis occurs [11,12]. To combat this clinical challenge, extensive research has been conducted across diverse dimensions of CRC oncology, ranging from translational platforms like patient-derived xenograft (PDX) models [13] to the identification of novel therapeutic compounds across various clinical stages [14]. Furthermore, innovative diagnostic approaches, such as radiomics-based computed tomography (CT) analysis to non-invasively predict critical molecular profiles (KRAS/NRAS/BRAF), are actively driving the field toward precision medicine [15]. Amidst these expanding multimodal strategies, defining precise prognostic trajectories remains paramount. However, patients with lung-limited metastases generally have a better prognosis than those with liver or other organ involvement [16]. Pulmonary metastasectomy (PM) for CRC has been widely performed based on retrospective case series showing favorable outcomes. The International Registry of Lung Metastases, analyzing over 5000 cases, reported 5-year survival rates ranging from 30% to 60% in selected patients [17]. However, most of the evidence supporting PM came from retrospective studies [18,19,20,21], and combined with ethical considerations, prospective trials evaluating the efficacy of PM had been virtually nonexistent. The PulMiCC trial, reported in 2019, was the first randomized controlled trial to compare the efficacy of resection for lung metastases from CRC [22]. While this trial failed to demonstrate a clear superiority of PM because of early termination and insufficient statistical power, it revealed that the 5-year survival rate in the group undergoing observation without PM was 29.6%, which was better than previously assumed [23,24]. This finding suggested that the prognostic benefit of PM may vary between patients, although the mechanism remains unclear. However, existing prognostic factors provide insufficient predictive accuracy for optimal patient selection. While disease-free interval (DFI), number of metastases, laterality, and carcinoembryonic antigen (CEA) levels have been associated with outcomes [25,26,27], these individual parameters lack the discriminatory power to reliably identify patients who will benefit from PM. The persistently high recurrence rates following complete resection reflect this limitation, highlighting the need for a comprehensive, multifactorial prognostic prediction system [28,29]. Recently, a comprehensive meta-analysis by Gkikas et al. analyzed over 13,000 patients who underwent PM for colorectal cancer and identified preoperative prognostic factors that were significantly associated with 5-year overall survival (OS) [30]. This landmark study provides a robust foundation for developing a multifactorial prognostic model. We hypothesized that integrating these meta-analysis-derived prognostic factors into a weighted scoring system could enable accurate risk stratification of patients with colorectal lung metastases. We used the database of the Metastatic Lung Tumor Study Group of Japan (METAL-J), which comprises one of the largest multicenter cohorts of patients undergoing PM in Japan [31,32,33,34], to develop and validate a prognostic scoring system based on the Gkikas meta-analysis [30]. By stratifying patients into distinct prognostic groups, this approach may inform decisions regarding adjuvant chemotherapy following PM. We further evaluated its clinical utility in treatment stratification for patients with colorectal lung metastases.

## 2. Materials and Methods

### 2.1. Ethics Statement

This multicenter retrospective study was conducted in accordance with the principles of the Declaration of Helsinki and approved by the Institutional Review Board of Teikyo University School of Medicine (Approval number: 25-058, approval date: 16 September 2025). Because of the retrospective nature of this study, the requirement for individual informed consent was waived. All patient data were de-identified prior to analysis to ensure confidentiality and all data extraction and statistical analyses were strictly conducted after receiving this formal institutional approval.

### 2.2. Study Design and Data Source

This multicenter retrospective cohort study used data from the METAL-J database. METAL-J was established in 1984 as a collaborative research network comprising thoracic surgery departments across Japan. The database prospectively collects standardized clinical, surgical, and follow-up data on all patients undergoing PM at participating institutions. For the current analysis, we identified patients who underwent PM for CRC between January 2010 and December 2019. This period was selected to ensure the availability of modern chemotherapy regimens across institutions.

### 2.3. Definitions

The eight preoperative prognostic factors used in the scoring system were defined and assessed by the multidisciplinary tumor board at each participating institution according to its local clinical practice standards, reflecting real-world variability. Complete resection (R0) was defined as a microscopically margin-negative resection confirmed by pathological examination. Tumor size referred to the maximum diameter of the largest pulmonary metastasis measured on preoperative computed tomography, with ≥2 cm applied as the cutoff. Intrathoracic lymph node metastasis was defined as hilar or mediastinal nodal involvement, diagnosed by preoperative imaging, intraoperative assessment, or pathological examination. Preoperative serum CEA was classified as elevated at ≥5 ng/mL, based on the clinically established upper limit of normal. Preoperative chemotherapy, history of liver or extrathoracic distant metastasis, multiple (≥2) pulmonary metastases, and bilateral disease followed their conventional clinical definitions. Specific imaging protocols, assessment timing, and diagnostic thresholds were determined by each institution’s standard practice.

### 2.4. Development of the Prognostic Scoring Model

We developed a scoring model based on the systematic review and meta-analysis by Gkikas et al., which identified prognostic factors significantly associated with 5-year survival following PM for CRC [30]. Eight factors were selected based on (1) clinical availability in routine preoperative assessment, (2) statistical significance (*p* < 0.05) in the meta-analysis, and (3) robust supporting evidence (≥5 contributing studies). Integer weights were assigned in proportion to the natural logarithm of the published hazard ratios (HRs), normalized so that the smallest coefficient (history of liver metastasis) corresponded to one point [35]. Following established methodology for risk-scoring systems, the magnitude of each coefficient was integrated with the quality of supporting evidence, including the number of studies, heterogeneity (I^2^), and the precision of confidence intervals (CIs) [36]. Although the HR for preoperative chemotherapy was 1.72 in the meta-analysis, its score was reduced from 2 points to 1 point. This factor was supported by only eight studies, notably fewer than the other factors, and although substantial heterogeneity (I^2^ > 40%) was present for several variables, we applied conservative rounding to avoid overstating the weight of a factor resting on a relatively thin evidence base. This variable was retained in the final model because the history of pre-metastasectomy chemotherapy is a critical clinical factor when evaluating the subsequent impact of adjuvant chemotherapy, and its pooled hazard ratio remained statistically significant. The total score ranged from 0 to 13 points, with higher scores indicating poorer prognosis (Table 1). A priori risk group cutoffs were defined using cumulative HRs: low risk for scores 0–2, intermediate risk for scores 3–5, and high risk for scores ≥ 6, corresponding to approximately twofold and fourfold increases in baseline mortality risk, respectively (Table 2). As detailed in Table 2, the cumulative HR for each score was derived as 1.30^S, where S denotes the total score, and 1.30 represents the HR of the reference factor (history of liver metastasis). Neither the weights nor the cutoffs were derived from or optimized using the METAL-J data.

### 2.5. Surgical Procedures

Surgical approach (video-assisted thoracoscopic surgery vs. open thoracotomy), extent of resection (wedge resection, segmentectomy, or lobectomy), and lymph node dissection were determined by the operating surgeon at each institution based on tumor location, size, and the patient’s cardiopulmonary function. All surgical details were prospectively recorded in the METAL-J database at the time of surgery.

### 2.6. Adjuvant Chemotherapy

The decision to administer adjuvant chemotherapy following PM, including the choice of regimen, timing, and duration, was made by the multidisciplinary team at each institution based on individual patient factors, institutional protocols, and the treating physician’s judgment. Information regarding adjuvant chemotherapy administration and regimen details was prospectively recorded in the METAL-J database. For this analysis, patients were stratified solely based on whether they received any adjuvant chemotherapy (yes/no), regardless of specific regimen, dose, or duration.

### 2.7. Follow-Up

Patient follow-up was conducted according to each institution’s standard clinical practice protocols. Survival status and disease recurrence data were prospectively collected and updated in the METAL-J database at 6-month intervals for patients remaining under active follow-up. Follow-up methods typically included clinical examination, tumor marker assessment, and imaging studies, though the specific frequency and modalities varied across institutions. OS was calculated from the date of PM to the date of death from any cause or the last follow-up. Recurrence-free survival (RFS) was calculated from the date of PM to the date of first documented recurrence, death, or the last follow-up, whichever occurred first. Patients alive without recurrence were censored at the date of last follow-up.

### 2.8. Statistical Analysis

Post-recurrence survival (PRS) was calculated as the difference between OS and RFS and was assessed exclusively in patients who experienced recurrence. Kaplan–Meier estimates and log-rank tests were used for survival analysis across risk groups and within each risk group stratified by adjuvant chemotherapy. Multivariable Cox proportional hazards regression incorporating all eight prognostic factors was performed to validate the meta-analytic weights in the METAL-J cohort. Model discrimination was evaluated using Harrell’s concordance index (C-index) with administrative censoring at 60 months, corresponding to the 5-year survival endpoint targeted by the Gkikas meta-analysis, with 1000-iteration bootstrap resampling for 95% CIs. Linearity of the continuous METAL-J score was assessed using restricted cubic splines with three knots, and the Wald test for nonlinearity was used to evaluate the log-linearity assumption.

Adjuvant chemotherapy effects were assessed within each risk group using Cox proportional hazards regression over the entire follow-up period without administrative censoring, to capture the full long-term treatment effect. We then explored whether the effect of adjuvant chemotherapy varied across risk strata. These interaction analyses were exploratory rather than pre-specified. They arose during the study. A formal interaction test (high-risk score and adjuvant chemotherapy) was performed to evaluate whether the treatment effect differed across risk groups. An extended model incorporating all three risk groups, with the low-risk group as the reference category, was also constructed to assess dose–response relationships. The interaction between the continuous METAL-J score and adjuvant chemotherapy was evaluated to confirm dose–response relationships. Factor-by-factor interaction analyses were performed for each of the seven evaluable prognostic factors (intrathoracic lymph node metastasis was excluded because of sparse data). All tests were two-sided, and *p* < 0.05 was considered statistically significant. To address potential treatment selection bias inherent in retrospective adjuvant chemotherapy comparisons, inverse probability of treatment weighting (IPTW) was performed. A logistic regression model incorporating age, sex, and all eight Gkikas prognostic factors was used to estimate the propensity score for receiving adjuvant chemotherapy. Stabilized weights were calculated as *w* = *p*/PS for treated patients and *w* = (1 − *p*)/(1 − PS) for untreated patients, where *w* represents the stabilized weight, PS is the estimated propensity score, and *p* denotes the marginal probability of treatment. Weights were trimmed at the 1st and 99th percentiles. Covariate balance before and after IPTW was assessed using standardized mean differences (SMDs), with SMD < 0.1 considered indicative of adequate balance, and visualized using a Love plot. IPTW-weighted Cox regression with robust variance estimation was performed for each risk group and for the interaction model. In our study design, these IPTW-adjusted models served as the primary analysis to rigorously control for baseline imbalances, including perioperative chemotherapy history. Conversely, the unadjusted Cox regression analyses were utilized as a supplemental sensitivity analysis to confirm the robustness of the primary IPTW-weighted findings.

## 3. Results

### 3.1. Patient Characteristics

**Figure 1:**
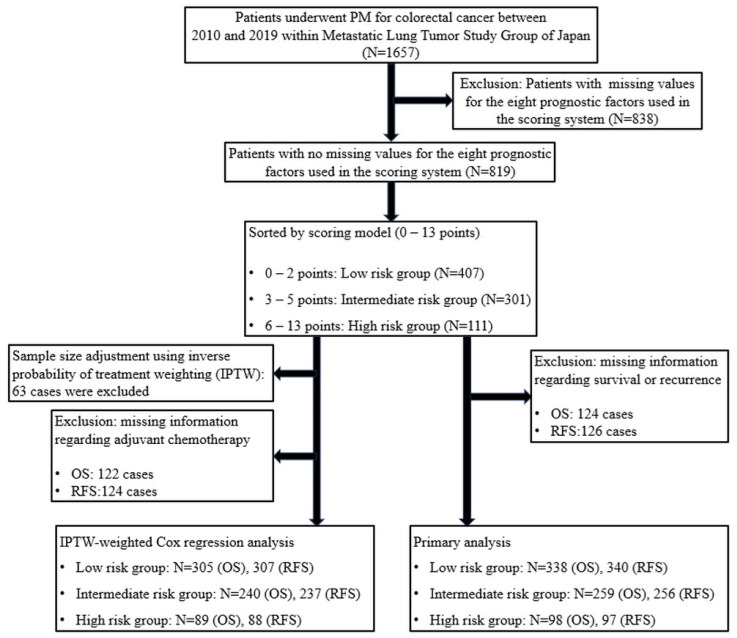
Screening flowchart. Abbreviations: PM = pulmonary metastasectomy, OS = overall survival, RFS = recurrence-free survival, IPTW = inverse probability of treatment weighting.

**Figure 2:**
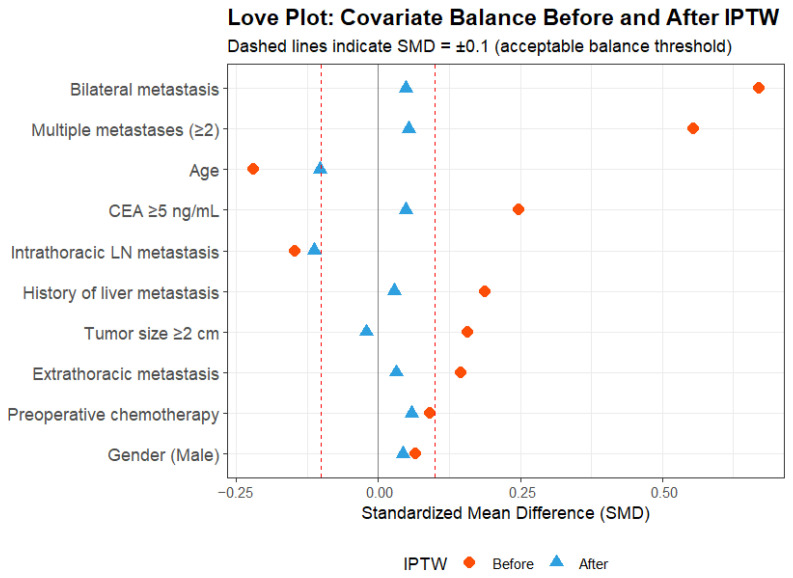
Love plot. Covariate balance before and after inverse probability of treatment weighting (IPTW). Standardized mean differences (SMDs) for each covariate are shown before (circles) and after (triangles) IPTW. Dashed lines indicate the SMD threshold of ±0.1 for acceptable balance.

**Figure A1:**
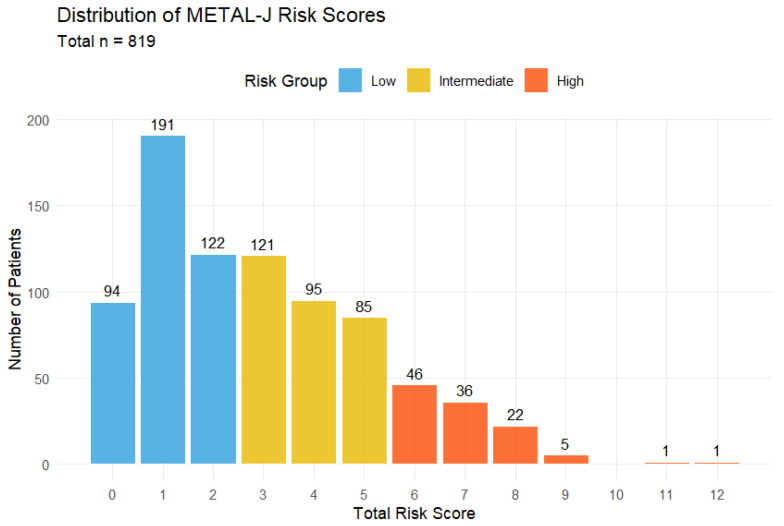
Histogram showing the number of cases for each score before IPTW adjustment.

Of the 1657 patients who underwent PM for CRC between January 2010 and December 2019, 819 patients (49.4%) with no missing data for all eight prognostic factors were included in the primary analysis (Figure A1). The median follow-up duration for survivors was 84 months (interquartile range [IQR]: 79–87). Based on the scoring model, the score ranged from 0 to 13 points with a median of 3 points (IQR: 1–4) (Figure A1), and patients were stratified into low-risk (*n* = 407), intermediate-risk (*n* = 301), and high-risk (*n* = 111) groups (Figure 1). Cases with missing values for survival (*n* = 124) or recurrence (*n* = 126) were excluded from the corresponding Kaplan–Meier analyses. Baseline characteristics of patients stratified by risk groups before and after IPTW adjustment are summarized in Table 3. Baseline demographics were similar across risk groups, with comparable distributions of age and primary tumor sites (rectum 55.0%, colon 43.0%) across groups. The eight scoring factors were distributed in line with the risk stratification (Table 3A). Of note, Stage IV disease at initial diagnosis, which was not incorporated into the scoring system, showed a clear gradient across groups (20.6%, 26.9%, and 38.7% in low-, intermediate-, and high-risk groups, respectively), consistent with the scoring system capturing broader disease aggressiveness beyond its constituent factors. Adjuvant chemotherapy after metastasectomy was administered to 6.9%, 16.9%, and 26.1% of patients in the low-, intermediate-, and high-risk groups, respectively. After IPTW adjustment, SMDs fell below 0.1 for all covariates except intrathoracic lymph node metastasis, for which residual imbalance reflected the small number of events (Figure 2, Table 3B).

### 3.2. Risk Group Stratification and Predictive Ability

**Figure 3:**
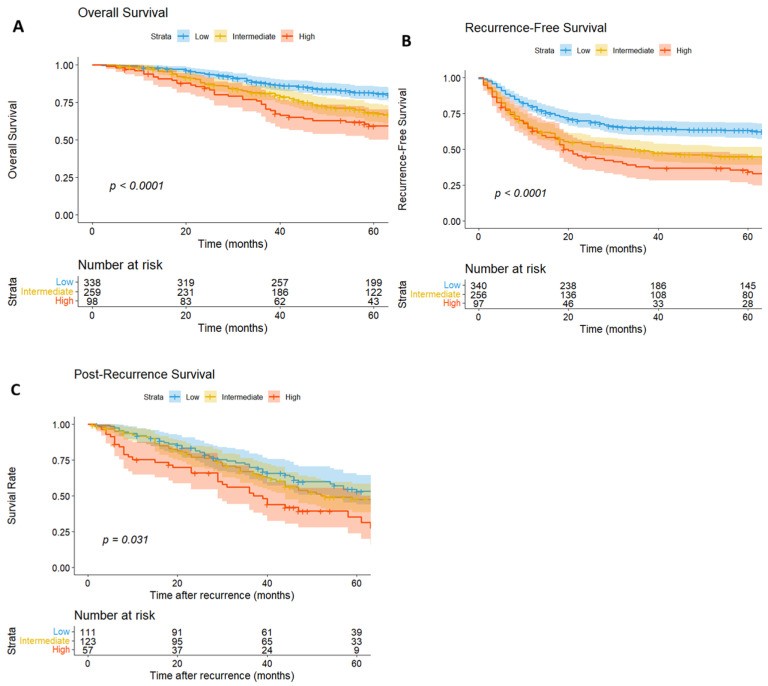
Kaplan–Meier curve for overall survival (**A**), recurrence-free survival (**B**), and post-recurrence survival (**C**). (**A**) 124 cases were excluded because of missing survival data. Log-rank test: low vs. intermediate *p* = 0.003, low vs. high *p* < 0.001, intermediate vs. high *p* = 0.033. (**B**) 126 cases were excluded because of missing recurrence data. Log-rank test: low vs. intermediate *p* < 0.001, low vs. high *p* < 0.001, intermediate vs. high *p* = 0.094. (**C**) The PRS analysis included 291 cases in which recurrence was observed after pulmonary metastasectomy Log-rank test: low vs. intermediate *p* = 0.646, low vs. high *p* = 0.010, intermediate vs. high *p* = 0.038.

Kaplan–Meier survival analysis demonstrated significant differences in outcomes among the three risk groups. The 5-year OS rates were 81.1% (95% CI: 76.7–85.6%) for the low-risk group, 67.8% (95% CI: 62.0–74.1%) for the intermediate-risk group, and 59.1% (95% CI: 49.9–70.1%) for the high-risk group. The 5-year RFS rates were 63.1% (95% CI: 58.1–68.6%), 44.8% (95% CI: 38.9–51.4%), and 34.2% (95% CI: 25.8–45.4%) for the low-, intermediate-, and high-risk groups, respectively. A log-rank test was performed on the OS data, revealing significant differences between groups, indicating stratification (low vs. intermediate: *p* = 0.003, low vs. high: *p* < 0.001, intermediate vs. high: *p* = 0.033; Figure 3A). For RFS, no significant difference was observed between the intermediate- and high-risk groups (*p* = 0.094), but significant differences were observed in the other combinations (low vs. intermediate: *p* < 0.001, low vs. high: *p* < 0.001; Figure 3B). The 5-year PRS rates were 53.1% (95% CI: 43.7–64.5%) for the low-risk group, 47.5% (95% CI: 38.5–58.4%) for the intermediate-risk group, and 35.3% (95% CI: 23.6–52.8%) for the high-risk group. For PRS, no significant difference was observed between the low- and intermediate-risk groups (*p* = 0.646), but significant differences were observed in the other combinations (low vs. high: *p* = 0.010, intermediate vs. high: *p* = 0.038; Figure 3C). To validate the meta-analytic weights in the METAL-J cohort, multivariable Cox regression incorporating all eight prognostic factors was performed. Six of eight factors showed concordant directionality with the Gkikas estimates (Table 4). The full eight-factor model achieved a C-index of 0.634, while the simplified integer-weighted METAL-J score achieved a C-index of 0.612 (95% CI: 0.565–0.657) for OS and 0.607 (95% CI: 0.576–0.635) for RFS, indicating that the score retained most of the discriminatory capacity of the full multivariable model despite its simplicity. Restricted cubic spline analysis showed no significant departure from log-linearity (*p* = 0.350).

### 3.3. The Effectiveness of Adjuvant Chemotherapy for Lung Metastases

**Figure 4:**
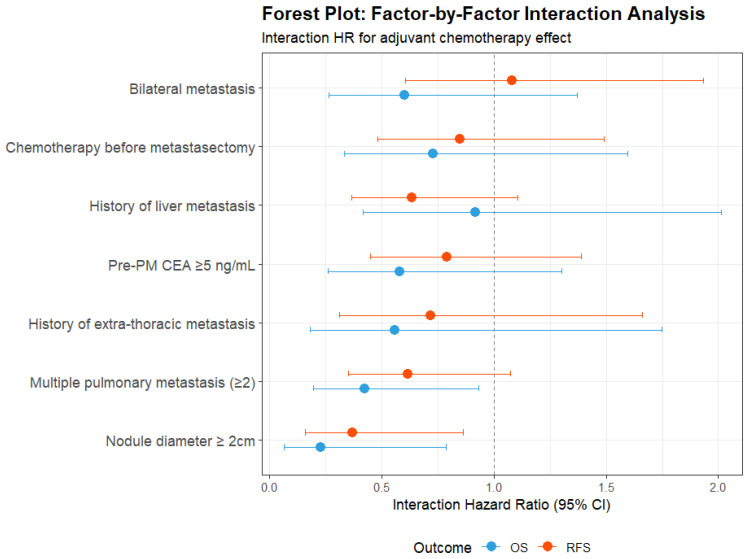
Forest plots of adjuvant chemotherapy interaction analyses. Factor-by-factor interaction analysis showing the interaction hazard ratio (HR) between each prognostic factor and adjuvant chemotherapy for overall survival (OS) and recurrence-free survival (RFS). HR < 1.0 indicates that the prognostic factor enhances adjuvant chemotherapy efficacy.

**Figure A2:**
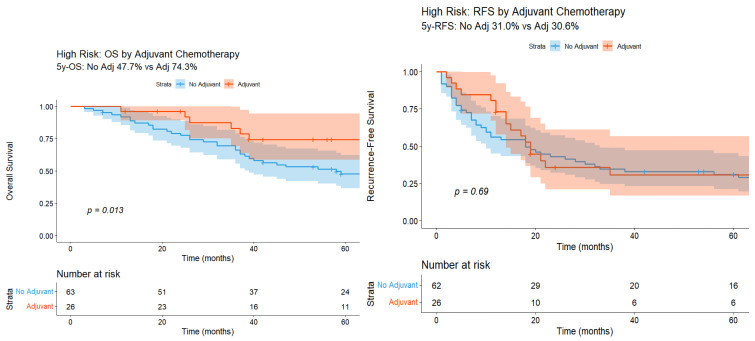
Kaplan–Meier curves for each risk group before IPTW adjustment, classified by the presence or absence of postoperative adjuvant chemotherapy following pulmonary metastasectomy.

Among 819 patients, adjuvant chemotherapy data were available for 759. Treatment effects varied markedly by risk group (Table 5). In the low-risk group, adjuvant chemotherapy showed no significant OS benefit (HR 1.71, 95% CI 0.88–3.31, *p* = 0.111), with a point estimate suggesting potential harm. In the intermediate-risk group, no significant OS benefit was observed (HR 0.65, 95% CI 0.36–1.17, *p* = 0.148). In the high-risk group, adjuvant chemotherapy was associated with a significant reduction in mortality risk (HR 0.35, 95% CI 0.15–0.83, *p* = 0.017; Figure A2). The overall unstratified analysis showed no significant OS benefit (HR 0.86, 95% CI 0.58–1.28, *p* = 0.460). For RFS, a significant adverse effect was observed in the low-risk group (HR 1.90, 95% CI 1.14–3.17, *p* = 0.014), while no significant RFS benefit was observed in the high-risk group (HR 0.89, 95% CI 0.52–1.55, *p* = 0.690). The additional interaction test demonstrated that the treatment effect of adjuvant chemotherapy differed significantly between high- and non-high-risk patients for OS (interaction HR 0.34, 95% CI 0.13–0.91, *p* = 0.031), but not for RFS (interaction HR 0.59, 95% CI 0.31–1.11, *p* = 0.104; Table 6A). In the extended three-group model with low-risk as the reference, the interaction term for adjuvant chemotherapy and the high-risk group was significant for both OS (interaction HR 0.20, 95% CI 0.07–0.60, *p* = 0.004) and RFS (interaction HR 0.45, 95% CI 0.21–0.96, *p* = 0.039; Table 6B), with a dose–response pattern in which increasing risk scores corresponded to progressively greater adjuvant chemotherapy benefit. The continuous score and adjuvant chemotherapy interaction confirmed this dose–response relationship: each 1-point increase in the score multiplied the adjuvant chemotherapy HR by 0.77 for OS (95% CI 0.65–0.91, *p* = 0.003) and 0.89 for RFS (95% CI 0.79–1.00, *p* = 0.043; Table 7). Among the seven assessable prognostic factors, nodule diameter ≥ 2 cm showed individually significant effect modification for both OS (interaction HR 0.23, 95% CI 0.07–0.79, *p* = 0.019) and RFS (interaction HR 0.37, 95% CI 0.16–0.86, *p* = 0.021). Multiple pulmonary metastases showed significant effect modification for OS (interaction HR 0.43, 95% CI 0.19–0.93, *p* = 0.032) with a borderline trend for RFS (*p* = 0.087; Table 8, Figure 4).

### 3.4. Sensitivity Analysis Using IPTW Adjustment

**Figure 5:**
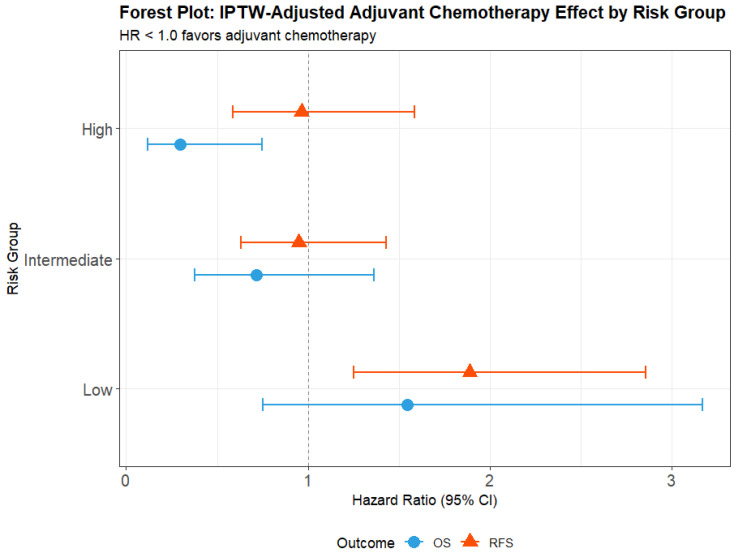
Forest plots of adjuvant chemotherapy interaction analyses. IPTW-adjusted adjuvant chemotherapy effect by risk group for OS and RFS. HR < 1.0 favors adjuvant chemotherapy. Error bars represent 95% confidence intervals.

**Figure 6:**
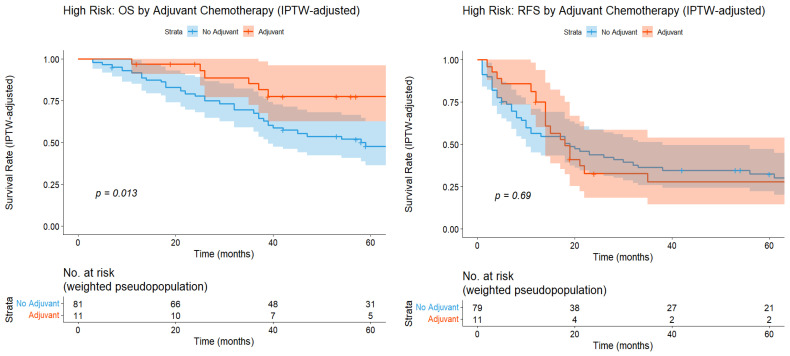

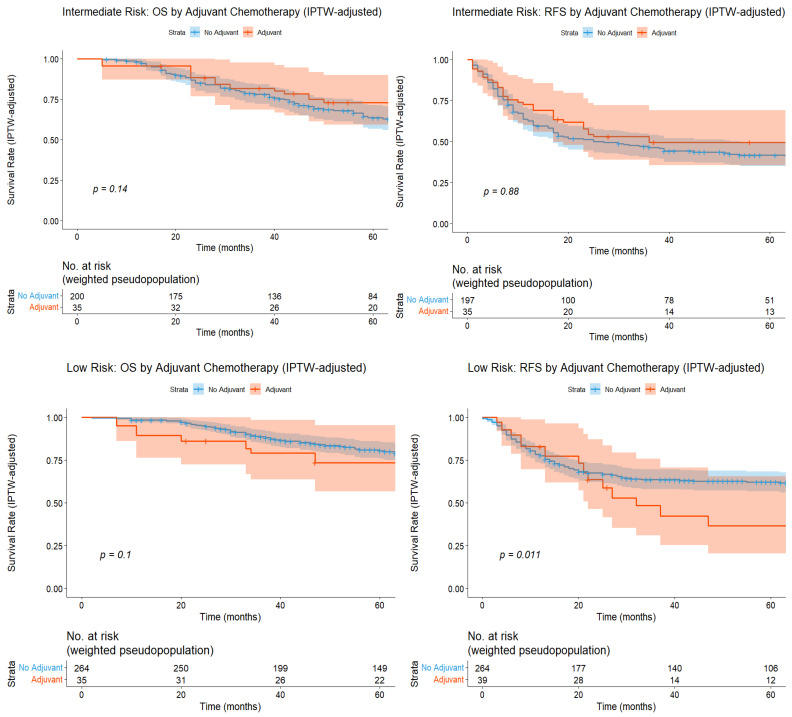
Kaplan–Meier curves for each risk group after IPTW adjustment, classified by the presence or absence of postoperative adjuvant chemotherapy following PM.

Among 756 patients with complete propensity score covariates, IPTW-weighted analysis confirmed the risk-stratified pattern (Table 3B, Figure 5, Table A3). After IPTW adjustment, the high-risk group retained a significant OS benefit from adjuvant chemotherapy, while the low- and intermediate-risk groups did not (Figure 6, Table 9). The IPTW-adjusted interaction test remained significant for OS (interaction HR 0.27, 95% CI 0.09–0.78, *p* = 0.015), providing evidence that the differential treatment effect is not attributable to measured confounding. For RFS, the IPTW-adjusted interaction was not significant (*p* = 0.181; Table 10), consistent with the unadjusted analysis.

## 4. Discussion

This study developed and validated a prognostic scoring system for patients undergoing PM for CRC that was systematically derived from eight prognostic factors identified in a comprehensive meta-analysis. The scoring system stratified patients into three distinct prognostic groups, with 5-year OS rates of 81.1%, 67.8%, and 59.1% for the low-, intermediate-, and high-risk groups, respectively. The score achieved moderate discriminatory ability with a C-index of 0.612 for OS and 0.607 for RFS, with administrative censoring at 5 years. Tumor size ≥ 2 cm and multiple metastases were identified as factors modifying the efficacy of adjuvant chemotherapy. In the low-risk group, adjuvant chemotherapy was associated with significantly worse RFS. This observation is hypothesis-generating and suggests that our data do not support a routine benefit of adjuvant chemotherapy in low-risk patients, although this requires prospective confirmation.

**Figure A3:**
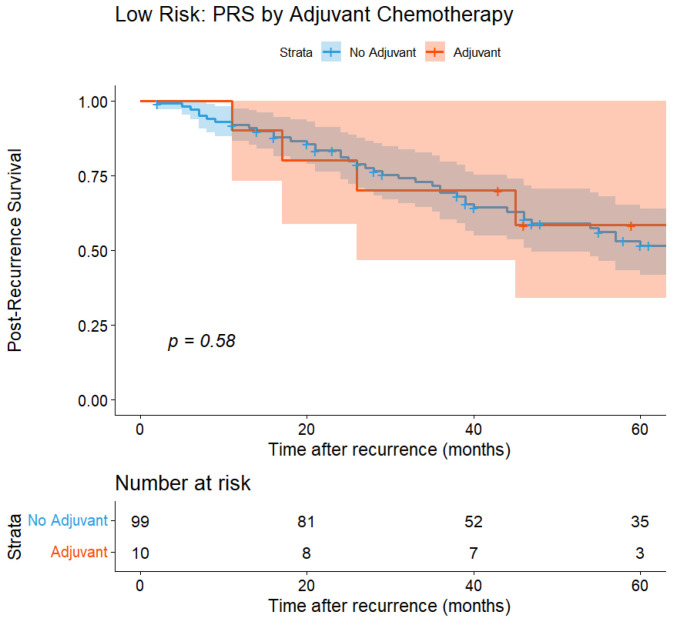
Kaplan–Meier curves for post-recurrence survival (PRS), classified by the presence or absence of post-metastasectomy adjuvant chemotherapy in each risk group (before IPTW adjustment). The number of patients at risk is shown beneath each curve. In the high-risk group, the PRS analysis included 16 patients who received and 39 who did not receive adjuvant chemotherapy.

However, the OS benefit observed in the high-risk group warrants careful interpretation, as several internal findings argue against a direct treatment effect of adjuvant chemotherapy. If adjuvant chemotherapy exerted a genuine biological effect on systemic micrometastases, a parallel benefit on RFS would be expected. In fact, no such benefit was observed in the high-risk group (HR 0.89, 95% CI 0.52–1.55, *p* = 0.690), either before or after IPTW adjustment. By contrast, the difference in PRS within the high-risk group was striking: patients who had received adjuvant chemotherapy had a 5-year PRS of 67.7%, compared with 20.3% in those who had not (*p* = 0.012; Figure A3, Table A2). The corresponding PRS findings, derived mathematically from OS and RFS, are presented in Figure A3 and Table A2 for completeness.

We therefore interpret the apparent OS benefit in the high-risk group not as a direct antitumor effect of perioperative systemic therapy, but as a marker of patient fitness for salvage treatment after recurrence. Patients selected for adjuvant chemotherapy in routine practice are, by definition, those judged by the multidisciplinary team to tolerate systemic therapy, reflecting preserved performance status, adequate organ function, and the absence of prohibitive comorbidities. These same characteristics predict the ability to receive intensive multidisciplinary treatment at the time of recurrence, including repeat metastasectomy, second- or later-line chemotherapy, radiation, and molecular-targeted agents, all of which are the primary determinants of PRS in this disease. Under this interpretation, adjuvant chemotherapy in the high-risk group acts as a selection marker for treatment-fit patients rather than a direct treatment effect, and IPTW cannot account for unmeasured confounding. This interpretation warrants validation in datasets incorporating detailed post-recurrence treatment information. Consistent with this view, the RFS-based interaction did not retain significance after IPTW (*p* = 0.181; Table 9).

The role of adjuvant chemotherapy following PM for CRC remains controversial, with conflicting evidence from recent studies. While a Japanese phase 2 trial (WJOG5810G) demonstrated promising outcomes with adjuvant mFOLFOX6 (5-year OS 85.2%, disease-free survival (DFS) 60.2%), a propensity score-matched analysis found no survival benefit, and a recent meta-analysis reported no OS improvement for lung metastases, though DFS benefit was observed for liver metastases [37,38,39]. These inconsistent findings likely reflect substantial patient heterogeneity, where potential benefits in selected patients may be obscured by a lack of benefit in others. Our analysis contributes to this debate by suggesting that the conflicting findings in prior reports may reflect differences in patient selection rather than true variability in chemotherapy efficacy. Neither the apparent OS benefit observed in the high-risk group nor the adverse effect on RFS in the low-risk group can be definitively interpreted as a true biological treatment effect of adjuvant chemotherapy. Given the retrospective nature of our database, these findings are highly susceptible to residual confounding by unmeasured variables—such as performance status, comorbidity burden, primary tumor molecular profiles (RAS/BRAF status), and primary tumor sidedness—which could not be adjusted for even after IPTW. Consequently, these observations should be viewed strictly as hypothesis-generating, and they do not justify definitive conclusions or immediate changes to routine clinical practice regarding post-PM chemotherapy.

The inverse pattern observed in the low-risk group merits separate consideration. In this group, adjuvant chemotherapy was associated with a significant adverse effect on RFS that persisted after IPTW adjustment. We interpret this signal not as a direct harmful biological effect of chemotherapy, but as a marker of unmeasured clinical concern. In routine clinical practice, adjuvant chemotherapy after PM is not administered to patients judged to be at a genuinely low risk of recurrence. The low-risk patients in our cohort who received adjuvant therapy are therefore likely to have harbored adverse features not captured by the eight scoring factors, such as aggressive molecular or pathological profiles at the primary site, short DFI, or borderline imaging findings, prompting the treating team to intensify therapy despite an apparently favorable score. Under this interpretation, the observed “harm” in the low-risk group reflects residual confounding by indication rather than a treatment effect, and the finding reinforces rather than undermines the scoring system.

Several prognostic models have previously been proposed. Salah et al. reported 5-year survival rates of 68.2%, 46.4%, and 26.1% for three risk groups identified using CEA, DFI, and the number of metastases [40]. Okumura et al. proposed a model that included five factors, including age, that identified three risk groups with survival rates of 89.4%, 72.5%, and 48.9% [41]. Ziranu et al. proposed the Meta-Lung Score with five factors, including primary tumor nodal status [42]. Similar to these studies, our model incorporates well-established prognostic factors, including CEA and the number of metastases.

Our model differs in several important aspects. First, whereas prior scoring models were developed within single cohorts without external validation, our model applies meta-analytic weights derived from over 13,000 patients and was validated in one of the world’s largest independent registries. Second, we incorporated intrathoracic lymph node involvement with the highest weight (3 points), reflecting its powerful adverse prognostic impact demonstrated across multiple studies but not systematically included in earlier models.

This study has several limitations that warrant consideration. First, this was a retrospective analysis of a selected patient population who underwent complete resection, which may introduce selection bias. The decision to proceed with PM was made by multidisciplinary teams at each institution based on clinical judgment, and patients with more aggressive disease characteristics may have been preferentially offered systemic therapy rather than surgery. Second, our analysis was limited to 819 patients (49.4%) of the original 1657 because of incomplete data for the eight prognostic factors. As shown in Table A1, the included and excluded populations differed substantially, particularly in the rates of perioperative chemotherapy. IPTW balanced covariates within the analyzed cohort but cannot address this selection at the point of inclusion. Therefore, this analytic cohort may represent a healthier or better-documented subset, which could limit the generalizability of our findings to the entire registry population. Third, the definitions and assessment of prognostic factors were determined by each participating institution according to its local clinical practice standards, which may have introduced some heterogeneity in how factors were evaluated and recorded. Fourth, information regarding specific chemotherapy regimens, timing, and duration was not consistently available, precluding detailed analysis of the optimal perioperative chemotherapy strategy. The registry lacked detailed data on performance status, immune status, and molecular biomarkers such as RAS/BRAF status and primary tumor sidedness. The absence of these variables underscores the risk of residual confounding, as discussed above. Fifth, intrathoracic lymph node metastasis, the highest-weighted factor, was diagnosed by preoperative imaging in most patients but by intraoperative or pathological assessment in others. This heterogeneity means the full score cannot always be applied preoperatively. A modified model restricted to imaging-based nodal assessment warrants evaluation in future studies. Sixth, the discriminatory capacity of our scoring system, with a C-index of 0.612 for OS, is modest, which implies a limited accuracy for individual-level prognostication. Nevertheless, the score remains highly useful and is better suited for group-level risk stratification, such as identifying target patient populations for future clinical trial designs. Seventh, the interaction analyses between risk groups and adjuvant chemotherapy were exploratory rather than pre-specified, and should be regarded as hypothesis-generating. Statistical power to detect treatment-effect modification was limited in some subgroups by small event counts. Factor-by-factor interaction could not be reliably assessed for intrathoracic lymph node metastasis because of sparse data, and this factor was excluded from that analysis. Accordingly, the absence of a statistically significant interaction in some subgroups may reflect limited power rather than a true absence of effect. Finally, because this study was conducted using a database from a single country, future prospective cohort studies, randomized controlled trials, or external validation using independent patient cohorts from different geographical regions and ethnicities are warranted to confirm the universal applicability and predictive performance of our scoring system.

## 5. Conclusions

By applying meta-analysis-derived prognostic weights to one of the largest independent PM cohorts reported to date, this study demonstrates that prognostic risk stratification meaningfully predicts post-resection survival in colorectal lung metastases. The framework is readily deployable from routinely available prognostic data and may support individualized surgical decision-making. Importantly, the differential association between adjuvant chemotherapy and outcomes across risk groups appears to reflect patient selection rather than a direct treatment effect. Given the established toxicity of systemic chemotherapy, routine adjuvant administration cannot be justified without demonstrable survival benefit. Our findings therefore suggest that routine use of adjuvant chemotherapy may not be beneficial for low-risk patients, and individualized decision-making considering clinical factors not captured by the score remains essential.

## Figures and Tables

**Table 1:** Scoring factors. In a meta-analysis published in 2023 by Gkikas et al. [30], items meeting the selection criteria described in the Methods section were adopted as scoring factors. The smallest hazard ratio (HR) among the selected factors (HR 1.30, history of liver metastasis) was assigned 1 point as the reference, with other factors weighted proportionally to ln(HR).

Scoring Factor	Yes	No	HR (95% CI)	No. of Studies	I^2^	Cochrane’s Q-Test	β ^†^
Chemotherapy before metastasectomy *	1	0	1.72 (1.18–2.52)	8	46.30%	0.071	0.54
History of liver metastasis	1	0	1.30 (1.12–1.50)	21	30.60%	0.091	0.26
History of extrathoracic metastasis	1	0	1.33 (1.15–1.55)	27	46.60%	0.005	0.29
Multiple pulmonary metastases	2	0	1.66 (1.52–1.82)	32	1.60%	0.441	0.51
Bilateral metastasis	2	0	1.59 (1.34–1.89)	22	23%	0.162	0.46
Nodule diameter ≥ 2 cm	1	0	1.37 (1.15–1.64)	14	43.10%	0.043	0.31
Pre-PM CEA ≥ 5 ng/mL	2	0	1.77 (1.55–2.01)	32	42.60%	0.006	0.57
Metastasis in intrathoracic lymph nodes	3	0	2.05 (1.79–2.35)	27	11.40%	0.294	0.72

* One point was deducted from the score of the factor “chemotherapy before metastasectomy” because of a small number of studies (*n* < 10) and high heterogeneity (I^2^ > 40%). ^†^ The regression coefficient (β) was calculated by taking the logarithm of the HR for each item, and points were assigned in proportion to this value.

**Table 2:** Score and cumulative hazard ratio. The scoring system was calculated by assigning integer weights proportional to the natural logarithm of the hazard ratios (HRs) reported by Gkikas et al. [30]. The cumulative HR for each score was derived as 1.30^S, where S denotes the total score, and 1.30 represents the HR of the reference factor (history of liver metastasis). Risk groups were defined a priori based on cumulative HR thresholds entirely independent of our cohort: low risk (scores 0–2, cumulative HR < 2.0), intermediate risk (scores 3–5, cumulative HR 2.0–3.71), and high risk (scores ≥ 6, cumulative HR ≥ 4.83), corresponding to approximately twofold and fourfold increases in baseline mortality risk, respectively.

Score	Cumulative HR
0	1
1	1.30
2	1.69
3	2.20
4	2.86
5	3.71
6	4.83
7	6.27
8	8.16
9	10.6
10	13.8
11	17.9
12	23.3
13	30.3

**Table 3:** Characteristics of patients before IPTW (**A**) and after IPTW (**B**).

**A**	**Overall (*n* = 819)**			
**Variable**	**Low (*n* = 407)**	**Intermediate (*n* = 301)**	**High (*n* = 111)**	***p*-Value ^†^**
Gender				0.073
Male	255 (62.7%)	207 (68.8%)	64 (57.7%)	
Female	152 (37.3%)	94 (31.2%)	47 (42.3%)	
Age (years) *	67.0 (60.0–74.0)	68.0 (59.0–74.0)	67.0 (57.0–72.0)	0.500
Primary site				0.400
Rectum	222 (54.5%)	175 (58.1%)	54 (48.6%)	
Colon	179 (44.0%)	120 (39.9%)	56 (50.5%)	
Unknown	6 (1.5%)	6 (2.0%)	1 (0.9%)	
Stage of colorectal cancer				<0.001
Stage IV	84 (20.6%)	81 (26.9%)	43 (38.7%)	
Others	311 (76.4%)	204 (67.8%)	63 (56.8%)	
Unknown	12 (3.0%)	16 (5.3%)	5 (4.5%)	
Chemotherapy before metastasectomy				<0.001
Yes	212 (52.1%)	201 (66.8%)	76 (68.5%)	
No	195 (47.9%)	100 (33.2%)	35 (31.5%)	
Unknown	0 (0%)	0 (0%)	0 (0%)	
History of liver metastasis				<0.001
Yes	92 (22.6%)	99 (32.9%)	57 (51.4%)	
No	315 (77.4%)	202 (67.1%)	54 (48.6%)	
Unknown	0 (0%)	0 (0%)	0 (0%)	
History of extrathoracic metastasis				<0.001
Yes	21 (5.2%)	34 (11.3%)	19 (17.1%)	
No	386 (94.8%)	267 (88.7%)	92 (82.9%)	
Unknown	0 (0%)	0 (0%)	0 (0%)	
Multiple metastases (≥2)				<0.001
Yes	15 (3.7%)	176 (58.5%)	106 (95.5%)	
No	392 (96.3%)	125 (41.5%)	5 (4.5%)	
Unknown	0 (0%)	0 (0%)	0 (0%)	
Bilateral metastasis				<0.001
Yes	0 (0%)	70 (23.3%)	90 (81.1%)	
No	407 (100%)	231 (76.7%)	21 (18.9%)	
Unknown	0 (0%)	0 (0%)	0 (0%)	
Nodule diameter ≥ 2 cm				<0.001
Yes	34 (8.4%)	74 (24.6%)	32 (28.8%)	
No	373 (91.6%)	227 (75.4%)	79 (71.2%)	
Unknown	0 (0%)	0 (0%)	0 (0%)	
Metastasis in intrathoracic lymph nodes				<0.001
Yes	0 (0%)	10 (3.3%)	12 (10.8%)	
No	407 (100%)	291 (96.7%)	99 (89.2%)	
Unknown	0 (0%)	0 (0%)	0 (0%)	
Pre-PM CEA ≥ 5 ng/mL				<0.001
Yes	23 (5.7%)	119 (39.5%)	80 (72.1%)	
No	384 (94.3%)	182 (60.5%)	31 (27.9%)	
Unknown	0 (0%)	0 (0%)	0 (0%)	
Adjuvant chemotherapy after PM				<0.001
Yes	28 (6.9%)	51 (16.9%)	29 (26.1%)	
No	348 (85.5%)	230 (76.4%)	73 (65.8%)	
Unknown	31 (7.6%)	20 (6.7%)	9 (8.1%)	
**B**	**Overall (*n* = 756)**			
**Variable**	**Low (*n* = 373)**	**Intermediate (*n* = 281)**	**High (*n* = 102)**	
Gender				
Male	234 (62.7%)	191 (68.0%)	59 (57.8%)	
Female	139 (37.3%)	90 (32.0%)	43 (42.2%)	
Age (years) *	67.0 (60.0–74.0)	68.0 (59.0–73.0)	67.0 (56.0–72.0)	
Primary site				
Rectum	206 (55.2%)	165 (58.7%)	49 (48.0%)	
Colon	163 (43.7%)	112 (39.9%)	53 (52.0%)	
Unknown	4 (1.1%)	4 (1.4%)	0 (0%)	
Stage of colorectal cancer				
Stage IV	76 (20.4%)	71 (25.3%)	42 (41.2%)	
Others	285 (76.4%)	195 (69.4%)	55 (53.9%)	
Unknown	12 (3.2%)	15 (5.3%)	5 (4.9%)	
Chemotherapy before metastasectomy				
Yes	188 (50.4%)	184 (65.5%)	68 (66.7%)	
No	185 (49.6%)	97 (34.5%)	34 (33.3%)	
Unknown	0 (0%)	0 (0%)	0 (0%)	
History of liver metastasis				
Yes	80 (21.4%)	91 (32.4%)	52 (51.0%)	
No	293 (78.6%)	190 (67.6%)	50 (49.0%)	
Unknown	0 (0%)	0 (0%)	0 (0%)	
History of extrathoracic metastasis				
Yes	18 (4.8%)	32 (11.4%)	13 (12.7%)	
No	355 (95.2%)	249 (88.6%)	89 (87.3%)	
Unknown	0 (0%)	0 (0%)	0 (0%)	
Multiple metastases (≥2)				
Yes	15 (4.0%)	169 (60.1%)	98 (96.1%)	
No	358 (96.0%)	112 (39.9%)	4 (3.9%)	
Unknown	0 (0%)	0 (0%)	0 (0%)	
Bilateral metastasis				
Yes	0 (0%)	68 (24.2%)	85 (83.3%)	
No	373 (100%)	213 (75.8%)	17 (16.7%)	
Unknown	0 (0%)	0 (0%)	0 (0%)	
Nodule diameter ≥ 2 cm				
Yes	32 (8.6%)	64 (22.8%)	32 (31.4%)	
No	341 (91.4%)	217 (77.2%)	70 (68.6%)	
Unknown	0 (0%)	0 (0%)	0 (0%)	
Metastasis in intrathoracic lymph nodes				
Yes	0 (0%)	9 (3.2%)	11 (10.8%)	
No	373 (100%)	272 (96.8%)	91 (89.2%)	
Unknown	0 (0%)	0 (0%)	0 (0%)	
Pre-PM CEA ≥ 5 ng/mL				
Yes	23 (6.2%)	109 (38.8%)	74 (72.5%)	
No	350 (93.8%)	172 (61.2%)	28 (27.5%)	
Unknown	0 (0%)	0 (0%)	0 (0%)	
Adjuvant chemotherapy after PM				
Yes	28 (7.5%)	51 (18.1%)	29 (28.4%)	
No	345 (92.5%)	230 (81.9%)	73 (71.6%)	
Unknown	0 (0%)	0 (0%)	0 (0%)	

* *n* (%); median (Q1–Q3), ^†^ Pearson’s Chi-squared test; Kruskal–Wallis rank sum test.

**Table 4:** Multivariable Cox proportional hazards regression incorporating all eight prognostic factors was performed to validate the meta-analytic weights in the METAL-J cohort. A total of 124 observations were excluded because of missing values.

Prognostic Factors	HR (95% CI)	*p*-Value
Chemotherapy before metastasectomy	0.95 (0.73–1.24)	0.718
History of liver metastasis	1.63 (1.25–2.13)	<0.001
History of extrathoracic metastasis	1.55 (1.06–2.27)	0.023
Multiple pulmonary metastases	1.53 (1.11–2.11)	0.010
Bilateral metastasis	0.82 (0.56–1.21)	0.319
Nodule diameter ≥ 2 cm	1.31 (0.95–1.81)	0.099
Pre-PM CEA ≥ 5 ng/mL	1.57 (1.19–2.07)	0.002
Metastasis in intrathoracic lymph nodes	1.91 (1.07–3.43)	0.030

**Table 5:** The efficacy of adjuvant chemotherapy in each group. Cox proportional hazards regression was used to evaluate the efficacy of adjuvant chemotherapy within each risk group.

	Overall Survival		Recurrence-Free Survival	
	HR (95% CI)	*p*-Value	HR (95% CI)	*p*-Value
Low-risk group	1.71 (0.88–3.31)	0.111	1.90 (1.14–3.17)	0.014
Intermediate-risk group	0.65 (0.36–1.17)	0.148	1.03 (0.68–1.56)	0.885
High-risk group	0.35 (0.15–0.83)	0.017	0.89 (0.52–1.55)	0.690
Overall	0.86 (0.58–1.28)	0.460	1.40 (1.06–1.84)	0.016

**Table 6:** (**A**) Interaction effect test. To assess whether treatment effects differed significantly among risk groups, a formal interaction test (high-risk score and adjuvant chemotherapy) was conducted. (**B**) To evaluate the dose–response relationship, an extended model incorporating all three risk groups, with the low-risk group as the reference group, was constructed.

**A**	**Overall Survival**		**Recurrence-Free Survival**	
	**HR (95% CI)**	***p*-Value**	**HR (95% CI)**	***p*-Value**
Non-high-risk group	1.02 (0.66–1.59)	0.906	1.47 (1.07–2.03)	0.018
High-risk group	2.30 (1.62–3.27)	<0.001	1.90 (1.38–2.60)	<0.001
Interaction	0.34 (0.13–0.91)	0.031	0.59 (0.31–1.11)	0.104
(adjuvant chemotherapy/high risk)				
**B**	**Overall Survival**		**Recurrence-Free Survival**	
	**HR (95% CI)**	***p*** **-Value**	**HR (95% CI)**	***p*** **-Value**
Adjuvant chemotherapy in low-risk group	1.74 (0.90–3.36)	0.101	1.92 (1.15–3.20)	0.012
Adjuvant chemotherapy in intermediate-risk group	0.65 (0.36–1.17)	0.148	1.03 (0.68–1.56)	0.885
Adjuvant chemotherapy in high-risk group	0.35 (0.15–0.83)	0.017	0.89 (0.52–1.55)	0.690
Intermediate-risk group without adjuvant chemotherapy	1.71 (1.25–2.35)	<0.001	1.88 (1.45–2.42)	<0.001
High-risk group without adjuvant chemotherapy	2.91 (1.98–4.27)	<0.001	2.49 (1.77–3.51)	<0.001
Interaction (adjuvant chemotherapy/intermediate risk)	0.36 (0.15–0.88)	0.025	0.54 (0.28–1.04)	0.065
Interaction (adjuvant chemotherapy/high risk)	0.20 (0.07–0.60)	0.004	0.45 (0.21–0.96)	0.039

**Table 7:** The interaction between the continuous METAL-J score and adjuvant chemotherapy was evaluated to confirm the dose–response relationships.

	Overall Survival		Recurrence-Free Survival	
	HR (95% CI)	*p*-Value	HR (95% CI)	*p*-Value
Score	1.22 (1.15–1.29)	<0.001	1.19 (1.13–1.25)	<0.001
Adjuvant chemotherapy	1.99 (0.94–4.22)	0.074	1.83 (1.05–3.17)	0.032
Interaction	0.77 (0.65–0.91)	0.003	0.89 (0.79–1.00)	0.043
(score/adjuvant chemotherapy)				

**Table 8:** Analysis of interactions between prognostic factors. For each of the seven assessable prognostic factors, we performed an interaction analysis using Cox proportional hazards regression.

	Overall Survival		Recurrence-Free Survival	
Prognostic Factors *	HR (95% CI)	*p*-Value	HR (95% CI)	*p*-Value
Chemotherapy before metastasectomy	0.73 (0.33–1.60)	0.430	0.85 (0.48–1.49)	0.568
History of liver metastasis	0.92 (0.42–2.01)	0.828	0.64 (0.36–1.11)	0.109
History of extrathoracic metastasis	0.56 (0.18–1.75)	0.318	0.72 (0.31–1.66)	0.440
Multiple pulmonary metastases	0.43 (0.19–0.93)	0.032	0.62 (0.35–1.07)	0.087
Bilateral metastasis	0.60 (0.27–1.37)	0.227	1.08 (0.60–1.93)	0.795
Nodule diameter ≥ 2 cm	0.23 (0.07–0.79)	0.019	0.37 (0.16–0.86)	0.021
Pre-PM CEA ≥ 5 ng/mL	0.58 (0.26–1.30)	0.187	0.79 (0.45–1.39)	0.413

* Excludes “metastasis in intrathoracic lymph nodes” because the data are sparse.

**Table 9:** Five-year overall survival and recurrence-free survival for each risk group after IPTW adjustment.

Risk Group	Status of Adjuvant Chemotherapy	Five-Year Overall Survival (95% CI)	Five-Year Recurrence FREE Survival (95% CI)
Low	Adjuvant chemotherapy	73.4% (56.5–95.3%)	36.5% (20.4–65.4%)
	No adjuvant chemotherapy	80.4% (75.6–85.6%)	62.1% (56.6–68.2%)
Intermediate	Adjuvant chemotherapy	73.0% (59.2–89.9%)	49.5% (35.5–69.0%)
	No adjuvant chemotherapy	63.6% (56.7–71.2%)	41.7% (35.1–49.5%)
High	Adjuvant chemotherapy	77.5% (62.5–96.0%)	27.9% (9.3–53.8%)
	No adjuvant chemotherapy	47.7% (36.4–62.5%)	32.4% (22.3–47.2%)

**Table 10:** Interaction effect test after IPTW adjustment. After IPTW adjustment, a formal interaction test (high-risk score and adjuvant chemotherapy) was conducted.

	Overall Survival		Recurrence-Free Survival	
	HR (95% CI)	*p*-Value	HR (95% CI)	*p*-Value
Non-high-risk group	1.09 (0.67–1.75)	0.736	1.39 (1.04–1.86)	0.028
High-risk group	2.28 (1.59–3.27)	<0.001	1.83 (1.32–2.54)	<0.001
Interaction	0.27 (0.09–0.78)	0.015	0.68 (0.39–1.20)	0.181
(adjuvant chemotherapy/high risk)				

## Data Availability

The data presented in this study are available upon reasonable request from the corresponding author. The data are not publicly available because of institutional policy.

## Abbreviations

PM	Pulmonary metastasectomy
CRC	Colorectal cancer
DFI	Disease-free interval
CEA	Carcinoembryonic antigen
METAL-J	Metastatic Lung Tumor Study Group of Japan
IPTW	Inverse probability of treatment weighting
OS	Overall survival
RFS	Recurrence-free survival
PRS	Post-recurrence survival
C-index	Concordance index
CI	Confidence interval
SMD	Standardized mean difference
IQR	Interquartile range
HR	Hazard ratio

## Appendix A

**Table A1:** Baseline characteristics of patients excluded from this study. Complete cases and incomplete cases were compared using *t*-tests for age, number of metastases, and maximum tumor diameter, and using Chi-square tests for other variables. Cases classified as “Unknown” were excluded from the tests.

	Overall (*n* = 1657)		
Variable	Complete (*n* = 819)	Incomplete (*n* = 838)	*p*-Value
Gender			0.007
Male	526 (64.2%)	483 (57.6%)	
Female	293 (35.8%)	355 (42.4%)	
Unknown	0 (0%)	0 (0%)	
Age (years)	67.0 (59.0–74.0)	66.0 (58.0–73.0)	0.200
Primary site			0.009
Rectum	451 (55.1%)	418 (49.9%)	
Colon	355 (43.3%)	390 (46.5%)	
Unknown	13 (1.6%)	30 (3.6%)	
Stage of colorectal cancer			<0.001
Stage IV	208 (25.4%)	204 (24.3%)	
Others	578 (70.6%)	520 (62.1%)	
Unknown	33 (4.0%)	114 (13.6%)	
Chemotherapy before metastasectomy			<0.001
Yes	489 (59.7%)	113 (13.5%)	
No	330 (40.3%)	74 (8.8%)	
Unknown	0 (0%)	651 (77.7%)	
History of liver metastasis			<0.001
Yes	248 (30.3%)	177 (21.1%)	
No	571 (69.7%)	500 (59.7%)	
Unknown	0 (0%)	161 (19.2%)	
History of extrathoracic metastasis			<0.001
Yes	74 (9.0%)	22 (2.6%)	
No	745 (91.0%)	810 (96.7%)	
Unknown	0 (0%)	6 (0.7%)	
Multiple metastases (≥2)			<0.001
Yes	297 (36.3%)	54 (6.4%)	
No	522 (63.7%)	96 (11.5%)	
Unknown	0 (0%)	688 (82.1%)	
Bilateral metastasis			<0.001
Yes	160 (19.5%)	29 (3.5%)	
No	659 (80.5%)	121 (14.4%)	
Unknown	0 (0%)	688 (82.1%)	
Nodule diameter ≥ 2 cm			<0.001
Yes	140 (17.1%)	161 (19.2%)	
No	679 (82.9%)	619 (73.9%)	
Unknown	0 (0%)	58 (6.9%)	
Metastasis in intrathoracic lymph nodes			<0.001
Yes	22 (2.7%)	18 (2.1%)	
No	797 (97.3%)	759 (90.6%)	
Unknown	0 (0%)	61 (7.3%)	
Pre-PM CEA ≥ 5 ng/mL			<0.001
Yes	222 (27.1%)	196 (23.4%)	
No	597 (72.9%)	513 (61.2%)	
Unknown	0 (0%)	129 (15.4%)	
Adjuvant chemotherapy after PM			<0.001
Yes	108 (13.2%)	29 (3.5%)	
No	651 (79.5%)	160 (19.1%)	
Unknown	60 (7.3%)	649 (77.4%)	

**Table A2:** Five-year post-recurrence survival classification based on whether adjuvant chemotherapy was administered after surgery in each risk group.

Risk Group	Status of Adjuvant Chemotherapy	Five-Year Post-Recurrence Survival
		(95% CI)
Low	Adjuvant	58.3% (34.0–100%)
	No adjuvant	51.6% (41.7–63.8%)
Intermediate	Adjuvant	61.6% (42.6–88.9%)
	No adjuvant	42.0% (32.4–54.6%)
High	Adjuvant	67.7% (47.9–95.7%)
	No adjuvant	20.3% (10.0–41.1%)

**Table A3:** The efficacy of adjuvant chemotherapy in each group after IPTW adjustment. Cox proportional hazards regression was used to evaluate the efficacy of adjuvant chemotherapy within each risk group after IPTW adjustment.

	Overall Survival		Recurrence-Free Survival	
	HR (95% CI)	*p*-Value	HR (95% CI)	*p*-Value
Low-risk group	1.55 (0.75–3.17)	0.235	1.89 (1.25–2.86)	0.003
Intermediate-risk group	0.72 (0.38–1.36)	0.311	0.95 (0.63–1.43)	0.805
High-risk group	0.30 (0.12–0.75)	0.01	0.96 (0.59–1.59)	0.888
Overall	0.89 (0.57–1.38)	0.601	1.29 (1.00–1.68)	0.054
